# Antioxidant and Hypolipidemic Activity of the Hydroethanolic Extract of* Curatella americana* L. Leaves

**DOI:** 10.1155/2016/9681425

**Published:** 2016-05-09

**Authors:** Rafael Henrique Oliveira Lopes, Luis Fernando Benitez Macorini, Katia Ávila Antunes, Priscilla Pereira de Toledo Espindola, Tamaeh Monteiro Alfredo, Paola dos Santos da Rocha, Zefa Valdivina Pereira, Edson Lucas dos Santos, Kely de Picoli Souza

**Affiliations:** School of Environmental and Biological Science, Federal University of Grande Dourados, Rodovia Dourados Ithaum, Km 12, 79804-970 Dourados, MS, Brazil

## Abstract

High levels of reactive oxygen species in the body and hyperlipidemia are key factors for the development of cardiovascular diseases such as atherosclerosis. The present study investigated the antioxidant and hypolipidemic activity of hydroethanolic extract of* Curatella americana* L. leaves (ExC). The antioxidant activity of ExC was assessed by 2,2-diphenyl-1-picrylhydrazyl free radical (DPPH) scavenging capacity and protection against hemolysis induced by 2,2′-azobis(2-amidinopropane) dihydrochloride (AAPH), followed by quantification of malondialdehyde (MDA). Wistar rats with hyperlipidemia induced by high-fructose diet (60%) were treated for 60 days with water, simvastatin (30 mg·Kg^−1^), ciprofibrate (2 mg·Kg^−1^), and ExC (200 mg·Kg^−1^). ExC revealed IC_50_ of 6.0 ± 0.5 *μ*g·mL^−1^, an intermediary value among positive controls used in the assay of DPPH scavenging capacity. At all concentrations (50 to 125 *μ*g·mL^−1^) and times (60 to 240 min) evaluated, ExC protected erythrocytes against AAPH-induced hemolysis, which was confirmed by lower MDA levels.* In vivo* tests showed a reduction of 34 and 45%, respectively, in serum concentration of cholesterol and triglycerides in hyperlipidemic rats treated with ExC, a similar effect compared to the reference drugs, simvastatin and ciprofibrate, respectively. Together, the results showed the antioxidant activity of ExC and its ability to improve the serum lipid profile in hyperlipidemic rats.

## 1. Introduction

Hyperlipidemia causes about 17 million deaths worldwide each year [1]; in addition, it is also a key factor for the development of heart and coronary diseases and atherosclerosis. Atherosclerosis is a chronic inflammatory disease triggered by multiple factors, with strong contribution of endothelial damage related to lipid peroxidation. This endothelial dysfunction increases the permeation of low-density lipoproteins (LDL) through the intima layer, resulting in oxidation and formation of atherosclerotic damage [2, 3]. In order to control this imbalance, the body has enzymatic and nonenzymatic antioxidant defense mechanisms [4] capable of preventing the deleterious effects of oxidation, inhibiting lipid peroxidation, free radicals scavenging, and maintaining redox balance in cells.

In addition to endogenous antioxidants, there are antioxidants from exogenous sources. The beneficial effects of foods have been linked to the presence of bioactive compounds and other nutrients. Examples of biomolecules that have antioxidant potential are phenolic compounds such as isoflavones, phenolic acids, catechins, chlorogenic acids, anthocyanins, and terpenes [5]. Thus, plants have been described as an alternative to the development of new drugs [6] applied to treatment of many diseases such as hypercholesterolemia, ulcers, depurative blood, and cancer [7–9].

*Curatella americana* L. is a member of the Dilleniaceae family, popularly known in Brazil as “lixa or lixeira” [10]. The beneficial effects of* C. americana* have been described in scientific research and indicated by its popular use. The anti-inflammatory, analgesic, antihypertensive, and vasodilator effects of the hydroethanolic extract of* Curatella americana* L. leaves have been evaluated in [11–13]. In folk medicine, leaf decoction is used as an antiseptic and astringent; bark infusion is used for the treatment of cold and healing wounds, ulcers, diabetes, and hypertension [14].

In this context, the aim of this study was to evaluate the antioxidant and hypolipidemic activity of the hydroethanolic extract of* Curatella americana* L. leaves (ExC) on rats with hyperlipidemia induced by high-fructose diet.

## 2. Material and Methods

### 2.1. Plant Material and Extract Preparation

*C. americana* L. leaves were collected in Mato Grosso do Sul, Brazil. The plant material was dried (45–50°C), crushed, and macerated in ethanol : water (80 : 20, v/v) at room temperature for seven days. After this period, the extract was filtered, concentrated in a rotary vacuum evaporator (FISATOM), and lyophilized. The lyophilized ExC was stored at 4°C and protected from light.

### 2.2. Dosage of Phenolic Compounds, Total Flavonoids, and Saponins

The concentration of phenolic compounds in samples was determined by the spectrophotometric method described by [15] using the Folin-Ciocalteu method. Three ExC measurements were performed, the average being presented in mg of gallic acid equivalents (GAE) per 100 g of sample.

The content of total flavonoids was determined according to methodology described by [16], with some adaptations, using 2% aluminum chloride solution in methanol as reagent. Extract solutions were prepared in methanol : water (1 : 1) at concentration of 10 mg·mL^−1^. About 0.5 mL ExC was added to 4.5 mL methanolic solution of 2% hydrate aluminum chloride. After 30 min at rest, the absorbance of solutions was read at 415 nm.

The presence of saponins was evaluated by preparing 10 mg of ExC dissolved in 2 mL of ethanol. Then, 5 mL of boiling water was added; the sample was vigorously shaken and allowed to stand for 20 min. According to [17], foaming indicates the presence of saponins.

### 2.3. 2,2-Diphenyl-1-picrylhydrazyl (DPPH) Free Radical Scavenging Activity

The antioxidant activity of the hydroethanolic extract of* C. americana* L. leaves was evaluated using technique described and adapted by [18] of 2,2-diphenyl-1-picrylhydrazyl (DPPH) free radical scavenging. DPPH solutions were prepared (0.11 mM) using ascorbic acid and butylhydroxytoluene (BHT) as positive controls and ExC at concentration of 20 mg·mL^−1^ in 80% ethanol solution. Serial dilutions were prepared based on these solutions in the concentrations investigated. To establish the half-maximal inhibitory concentration (IC_50_) of DPPH free radical scavenging, the samples were tested in serial dilutions (0.1, 1, 5, 10, 25, 50, 100, 500, and 1000 *μ*g/mL) and analyzed by means of nonlinear regression using the Prism 5 GraphPad Software. Samples were assessed by spectrophotometer at 517 nm. The absorbance of each sample was divided by the absorbance of DPPH and multiplied by one hundred to represent the antioxidant activity in percentage. All independent experiments were performed in triplicate.

### 2.4. Protection against Hemolysis Induced by 2,2′-Azobis(2-amidinopropane) Dihydrochloride (AAPH)

Protection against lipid peroxidation of the extract was evaluated by hemolysis technique induced by 2,2-azobis 2-amidinopropane dihydrochloride (AAPH), described in [19]. About 5 mL of peripheral blood was collected from healthy donors, stored in tubes with sodium citrate (protocol approved by Ethics Research Committee: protocol number 123/12), and subsequently centrifuged at 2000 rpm for 5 min. The buffy coat was removed from plasma. The remaining erythrocytes underwent three washes with saline (0.9% NaCl) at 1500 rpm to remove possible interferences, with the supernatant discarded after each washing cycle. Subsequently, a 10% erythrocyte solution was prepared in saline.

The erythrocyte solution was incubated with distilled water (total hemolysis) and hemolysis induced by 2,2′-azobis(2-amidinopropane) dihydrochloride (AAPH; Sigma-Aldrich®) (50 mM) alone or concomitant with the standard antioxidant, ascorbic acid (AA), and ExC at concentrations of 50, 75, 100, and 125 *µ*g·mL^−1^, reaching a final concentration of red blood cells of 2.5%. Aliquots were taken every 60 min after the start of incubation for 240 min, which were read in spectrophotometer at 540 nm. Three independent experiments were performed in duplicate.

### 2.5. Dosage of Malondialdehyde (MDA)

A 20% suspension was used to assess the protective effects of ExC against lipid peroxidation. The dosage of MDA was evaluated after 240 min of incubation at 37°C with and without addition of 500 *µ*L of peroxyl radicals generated by thermal decomposition of 50 mM AAPH diluted in saline (0.9% NaCl). For this, after the incubation period, an aliquot of 0.5 mL of the reaction mixture was collected and was added to 0.5 mL of 20% trichloroacetic acid with subsequent homogenization. An aliquot of 0.5 mL was removed from this mixture and added into tubes previously pipetted with 1 mL of thiobarbituric acid reagent (TBA) 10 nM and incubated in water bath at 94°C for 45 min. After this period, samples were kept at room temperature for 15 min, followed by addition of 3 mL of butanol with subsequent agitation and centrifugation as described by [20]. Reading of the supernatant absorbance was carried out in spectrophotometer at 532 nm. Three independent experiments were performed in duplicate.

### 2.6. Hyperlipidemia Induced by High-Fructose Diet

The experimental procedures were approved by the Ethics Research Committee on Animal Experiments of UFGD under protocol number 022/2012. Wistar rats weighting approximately 156 ± 9 g were pretreated for 90 days with high-fructose diet (66%), prepared with 330 g of commercial chow (Labina) mixed with 660 g of fructose to induce hyperlipidemia. Concomitantly, normoglycemic rats were kept in commercial rodent chow (Labina) during all experimental period constituting the control group (control diet group, CD). All rats were kept in controlled light cycle and temperature with feed and water being offered* ad libitum*.

### 2.7. Experimental Design

Normoglycemic (*n* = 5) and hyperlipidemic Wistar rats (28) were assessed in this study. Hyperlipidemic rats were divided into four groups (*n* = 7 each) and daily provided by gavage for 60 days of water (control), simvastatin (30 mg·Kg^−1^ of body weight, simvastatin group), ciprofibrate (2 mg·Kg^−1^ of body weight, ciprofibrate group), and ExC (200 mg·Kg^−1^ of body weight, ExC group). At the end of treatment and after euthanasia, organs, tissue, and blood were collected for analysis.

### 2.8. Biochemical Analysis

The blood collected was centrifuged at 3000 rpm for 10 min and serum was used to measure total cholesterol, HDL-cholesterol, triglycerides, aminotransferases (AST and ALT), urea, and creatinine with support of Integra 400 Plus equipment (Roche*™*).

### 2.9. Statistical Analysis

Data are shown as mean ± standard error of the mean and were submitted to one-way analysis of variance (ANOVA) followed by Tukey posttest. The results were considered significant when *P* < 0.05.

## 3. Results

### 3.1. Chemical Profile

The concentration of total phenolic compounds and flavonoids was 391 ± 5.0 mg EAG·100 g^−1^ of ExC and 59 ± 3.6 mg EQ·100 g^−1^ of ExC, and analyses were positive for saponins.

### 3.2. Antioxidant Activity

Considering the presence of potentially antioxidant substances in ExC, an* in vitro* evaluation of DPPH free radical scavenging at different concentrations was performed. The 50% inhibitory concentration (IC_50_) and the maximum activity in assay of DPPH free radical scavenging of ExC were approximately one-third that of BHT and three times higher than that of ascorbic acid as shown in Table 1.

**Figure 1:**
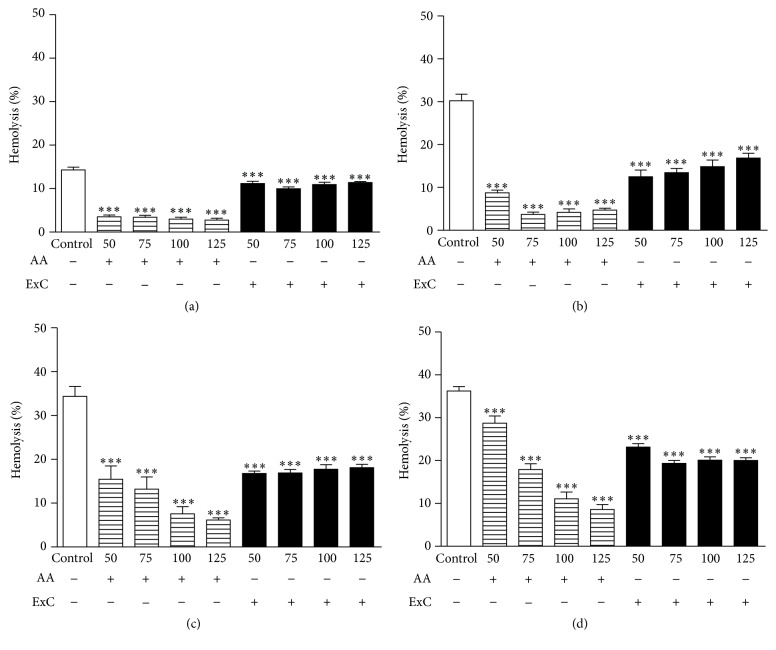
Hemolysis assessment at (a) 60, (b) 120, (c) 180, and (d) 240 min after addition of AAPH in erythrocytes at 2.5% (control) incubated with different concentrations (50–125 *µ*g·mL^−1^) of ascorbic acid (AA) and hydroethanolic extract of* C. americana* L. leaves (ExC). ^*∗∗∗*^ *P* < 0.0001 versus control samples.

The antihemolytic potential of ExC was evaluated in erythrocytes submitted to lipid peroxidation and consequent hemolysis induced by 2,2′-azobis(2-amidinopropane) dihydrochloride (AAPH) for 240 min at different concentrations. ExC decreased the hemolysis of erythrocytes induced by AAPH in a time dependent manner, but independent of evaluated dose as shown in Figure 1.

**Figure 2:**
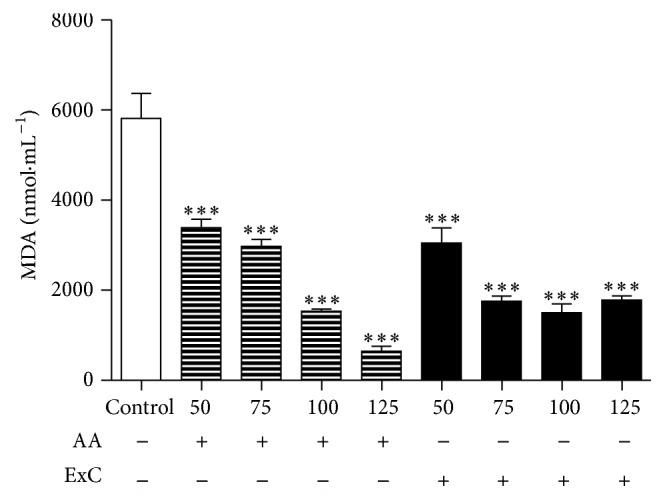
Malondialdehyde (MDA) concentration at 240 min after the addition of AAPH hemolysis inducer in 2.5% erythrocytes incubated with different concentrations (50–125 *µ*g·mL^−1^) of ascorbic acid (AA) and hydroethanolic extract of* C. americana* L. leaves (ExC) compared with control samples. ^*∗∗∗*^ *P* < 0.001 versus control samples.

Thus, the release of malonaldehyde (MDA) that occurs during the lipid peroxidation process was assessed. Erythrocytes incubated with ExC for 240 min at 125 *µ*g·mL^−1^ presented reduced MDA concentrations compared with control sample (*P* < 0.001) and similar to samples incubated with ascorbic acid (Figure 2).

### 3.3. Hypolipidemic Effect

Rats treated with ExC showed decreased serum levels of total cholesterol and triglycerides, 34% and 45%, respectively, compared to control hyperlipidemic rats. Similar results were observed for the standard drugs, ciprofibrate, used to control cholesterol, and simvastatin, used to control triglycerides. Other biochemical parameters evaluated regarding the hepatic and renal functions were similar among groups investigated (Table 2). However, the analysis between hyperlipidemic and normolipidemic rats (*t*-test analyses) has shown an increase in the serum levels of AST in both groups treated with simvastatin and ciprofibrate, which did not occur in ExC group.

## 4. Discussion

The aim of this study was to evaluate the antioxidant and hypolipidemic activities of the hydroethanolic extract of* C. americana* L. leaves related to both human health conditions and interest in the development of new drugs.

The oxidative balance in the body is regulated by endogenous and exogenous mechanisms, in which the excess of free radicals is related to many diseases [21]. The control of the excess of oxidative molecules includes especially exogenous intake of antioxidant molecules, which are largely found in plants [22]. The chemical composition of these plants has shown that same classes of polyphenols can exert such function such as flavonoids [23, 24]. The capacity of ExC of DPPH free radicals scavenging was intermediary among standard antioxidants and approximately three times higher than that of BHT. It is noteworthy that both controls used are isolated molecules, which stimulates new studies for the isolation of compounds from ExC, in which the presence of phenolic compounds and flavonoids was identified. In [25], investigating* Dillenia suffruticosa*, a plant of the Dilleniaceae family, identified the presence of polyphenols, although in lower amounts than those observed for* C. americana* L., and these compounds were correlated with antioxidant activity. In addition, another compound common to these two plants is saponin, which has shown hypocholesterolemic and anti-inflammatory activities; however, it has also been described to be able to promote destabilization of the cell membrane and induce hemolysis due to its emulsifying action [26–31], which was not observed for ExC at* in vivo* and* in vivo* studies.

ExC decreased lipid peroxidation induced by AAPH, protecting erythrocytes from cell death similarly to ascorbic acid, which is a vitamin with antioxidant capacity as demonstrated by decreased lipid peroxidation and malonaldehyde (MDA) production [32]. The lower levels of MDA produced during hemolysis induced by AAPH in samples incubated with ExC confirm the antioxidant action, also corroborated by [33] in the study of antioxidant activity of* Toona sinensis* leaves. The damage to the cell membrane resulting from lipid peroxidation induced by reactive species occurs in many diseases such as atherosclerosis, obesity, diabetes, hypertension, and cancer [21].

The importance of new products in the treatment and prevention of dyslipidemias becomes essential to reduce the mortality and morbidity due to cardiovascular complications. In addition, the search for less toxic drugs has increased the interest of the scientific community for natural products. The ExC showed to able to manage hyperlipidemia induced by high-fructose diet, reducing serum levels of total cholesterol and triglycerides, without signs of change in hepatic and renal function, suggesting that ExC is safe in the evaluated conditions. Ciprofibrate is a drug widely used to control cholesterol; however, it is contraindicated in patients with renal and hepatic disorders [34]. In this study, the ciprofibrate group showed an increase in liver and kidney weight (data not show), although the serum levels of ALT, creatinine, and urea remained unchanged. When compared to normolipidemic rats, the hyperlipidemic group treated with ciprofibrate presented an increase in serum level of AST.

The hypolipidemic activity of natural products can be correlated to the presence of flavonoids due to their properties of inhibiting cholesterol biosynthesis and absorption and modifying the activity of lipogenic and lipolytic enzymes, leading to reduced lipid metabolism [35–37], as observed in hyperlipidemic rats treated with ExC, which showed significant reduction in the levels of total cholesterol and triglycerides. Other molecules able to decrease the serum level of cholesterol are saponins [38], also present in ExC. It is very interesting that ExC was able to decrease both serum level of cholesterol and total triglycerides.

In conclusion, our results showed that* Curatella americana* L. leaves reduce oxidative stress by free radical scavenging and protect against lipid peroxidation and is also able to manage hyperlipidemia by decreasing serum level of cholesterol and triglycerides, similarly to standard drugs.

## Figures and Tables

**Table 1:** IC_50_ and maximum activity of DPPH free radical scavenging of standard antioxidants and the hydroethanolic extract of *C. americana *L. leaves (ExC).

Treatment	IC_50_ (*µ*g·mL^−1^)	*n* ^*∗*^	Maximum activity	
			%	*µ*g·mL^−1^
Ascorbic acid	1.8 ± 0.4	2	92.3 ± 0.8	10
BHT	18.3 ± 4.5	2	93.7 ± 1.3	500
ExC	6.0 ± 0.5	3	96.5 ± 1.2	25

^*∗*^ *n* = number of independent experiments in triplicate. BHT = butylhydroxytoluene.

**Table 2:** Serum lipid profile and hepatic and renal parameters of normolipidemic and hyperlipidemic Wistar rats induced by high-fructose diet (66%) treated with water (control), simvastatin (20 mg·kg^−1^ of body weight, simvastatin), ciprofibrate (2 mg·kg^−1^ of body weight, ciprofibrate), and hydroethanolic extract of *C. americana *L. leaves (ExC) (200 mg·kg^−1^ of body weight, ExC).

Parameters	Normolipidemic	Hyperlipidemic			
		Control	Simvastatin	Ciprofibrate	ExC
Total cholesterol (mg·dL^−1^)	80.0 ± 2.0^a^	118.3 ± 13.0^b^	91.3 ± 5.2^ab^	86.4 ± 4.3^a^	77.9 ± 4.1^a^
HDL-cholesterol (mg·dL^−1^)	45.0 ± 2.4^a^	58.0 ± 4.4^b^	54.0 ± 3.1^a^	47.0 ± 2.4^a^	46.0 ± 2.3^a^
Triglycerides (mg·dL^−1^)	138.3 ± 23.0^a^	225.0 ± 33.0^b^	119.0 ± 7.0^a^	155.0 ± 14.0^ab^	136.9 ± 7.5^a^
AST (U·L^−1^)	174.0 ± 6.5	178.0 ± 14.5	220.0 ± 20.1	209.0 ± 9.4	191.8 ± 12.3
ALT (U·L^−1^)	53.2 ± 3.3	65.4 ± 7.0	64.9 ± 5.6	65.0 ± 4.0	64.3 ± 9.3
Urea (U·L^−1^)	23.0 ± 2.0	30.0 ± 3.1	21.0 ± 1.4	26.0 ± 3.7	24.0 ± 3.8
Creatinine (U·L^−1^)	0.28 ± 0.02	0.34 ± 0.03	0.32 ± 0.02	0.30 ± 0.02	0.27 ± 0.02

Mean values followed by different superscript letters indicate significant difference (*P* < 0.05).
